# Supplementary material for: Hot spot prediction in protein-protein interactions by an ensemble system
Source: BMC Syst Biol. 2018 Dec 31;12(Suppl 9):132. doi: 10.1186/s12918-018-0665-8 (PMC6311905; doi:10.1186/s12918-018-0665-8)
Supplement: Supplementary file 2 — All relevant MATLAB code. A simple Matlab implement of our predictor. (ZIP 19,742 kb) [file 12918_2018_665_MOESM2_ESM.zip › Additional file 2/Read ASA/ASA website/ASEdb/1BXIA.htm]

 NetSurfP - Protein Surface Accessibility and Secondary Structure Predictions

  

|  |  |  |
| --- | --- | --- |
|  |  | NetSurfP - Protein Surface Accessibility and Secondary Structure Predictions  Technical University of Denmark |

  


---

```
# For publication of results, please cite:
# A generic method for assignment of reliability scores applied to solvent accessibility predictions.
# Bent Petersen, Thomas Nordahl Petersen, Pernille Andersen, Morten Nielsen and Claus Lundegaard
# BMC Structural Biology 2009, 9:51 doi:10.1186/1472-6807-9-51
#
# Column 1: Class assignment - B for buried or E for Exposed - Threshold: 25% exposure, but not based on RSA
# Column 2: Amino acid
# Column 3: Sequence name
# Column 4: Amino acid number
# Column 5: Relative Surface Accessibility - RSA
# Column 6: Absolute Surface Accessibility
# Column 7: Z-fit score for RSA prediction
# Column 8: Probability for Alpha-Helix
# Column 9: Probability for Beta-strand
# Column 10: Probability for Coil
E M  1BXIA                  1    0.665 133.107  -1.079   0.003   0.003   0.994
E E  1BXIA                  2    0.639 111.633   0.952   0.191   0.086   0.723
E L  1BXIA                  3    0.284  52.074  -0.498   0.278   0.093   0.628
E K  1BXIA                  4    0.423  87.073   0.818   0.455   0.046   0.498
E A  1BXIA                  5    0.580  63.894   0.273   0.430   0.016   0.555
E S  1BXIA                  6    0.463  54.310   1.107   0.430   0.016   0.555
B I  1BXIA                  7    0.129  23.791   0.034   0.430   0.016   0.555
E S  1BXIA                  8    0.479  56.104  -0.170   0.430   0.016   0.555
E D  1BXIA                  9    0.488  70.278  -0.158   0.430   0.016   0.555
B Y  1BXIA                 10    0.201  42.996   0.862   0.430   0.016   0.555
E T  1BXIA                 11    0.320  44.329   0.484   0.181   0.016   0.803
B E  1BXIA                 12    0.255  44.548   0.347   0.923   0.002   0.076
E A  1BXIA                 13    0.612  67.475   0.427   0.970   0.001   0.030
E E  1BXIA                 14    0.317  55.380   1.325   0.970   0.001   0.030
B F  1BXIA                 15    0.031   6.242   0.551   0.988   0.000   0.012
B L  1BXIA                 16    0.173  31.731   1.393   0.988   0.000   0.012
E Q  1BXIA                 17    0.487  87.032   1.467   0.988   0.000   0.012
B L  1BXIA                 18    0.075  13.659   0.636   0.988   0.000   0.012
B V  1BXIA                 19    0.028   4.242   0.672   0.988   0.000   0.012
E T  1BXIA                 20    0.397  55.036   1.616   0.988   0.000   0.012
E T  1BXIA                 21    0.406  56.354   1.859   0.970   0.001   0.030
B I  1BXIA                 22    0.034   6.234   0.237   0.975   0.003   0.022
B C  1BXIA                 23    0.147  20.639   0.955   0.938   0.007   0.055
E N  1BXIA                 24    0.611  89.436   1.424   0.782   0.003   0.216
E A  1BXIA                 25    0.400  44.091  -0.656   0.321   0.003   0.675
E D  1BXIA                 26    0.636  91.648  -1.274   0.053   0.005   0.942
E T  1BXIA                 27    0.426  59.100  -0.269   0.053   0.005   0.942
E S  1BXIA                 28    0.627  73.543   0.058   0.109   0.005   0.886
E S  1BXIA                 29    0.357  41.887  -0.306   0.109   0.005   0.886
E E  1BXIA                 30    0.482  84.275  -0.253   0.694   0.003   0.303
E E  1BXIA                 31    0.665 116.228   0.628   0.858   0.002   0.139
B E  1BXIA                 32    0.259  45.247   0.776   0.923   0.002   0.076
B L  1BXIA                 33    0.064  11.755   0.155   0.970   0.001   0.030
E V  1BXIA                 34    0.419  64.385   0.288   0.970   0.001   0.030
E K  1BXIA                 35    0.504 103.570   1.588   0.970   0.001   0.030
B L  1BXIA                 36    0.027   5.017   0.734   0.970   0.001   0.030
B V  1BXIA                 37    0.063   9.668   0.751   0.970   0.001   0.030
E T  1BXIA                 38    0.486  67.478   1.452   0.970   0.001   0.030
B H  1BXIA                 39    0.205  37.217   1.514   0.970   0.001   0.030
B F  1BXIA                 40    0.023   4.656   1.141   0.970   0.001   0.030
E E  1BXIA                 41    0.381  66.578   1.346   0.923   0.002   0.076
E E  1BXIA                 42    0.455  79.401   1.636   0.938   0.007   0.055
B M  1BXIA                 43    0.043   8.644   0.842   0.802   0.014   0.185
B T  1BXIA                 44    0.155  21.485   0.237   0.561   0.047   0.393
E E  1BXIA                 45    0.517  90.233   0.227   0.191   0.086   0.723
B H  1BXIA                 46    0.163  29.686   1.128   0.018   0.019   0.964
B P  1BXIA                 47    0.230  32.580  -0.139   0.018   0.019   0.964
E S  1BXIA                 48    0.626  73.309  -0.387   0.018   0.019   0.964
E G  1BXIA                 49    0.395  31.102  -1.565   0.018   0.019   0.964
E S  1BXIA                 50    0.337  39.520  -1.192   0.018   0.019   0.964
B D  1BXIA                 51    0.222  32.062  -1.169   0.004   0.085   0.910
B L  1BXIA                 52    0.044   7.965   0.464   0.004   0.616   0.381
B I  1BXIA                 53    0.043   7.918   0.187   0.002   0.816   0.182
B Y  1BXIA                 54    0.073  15.536   0.035   0.002   0.816   0.182
B Y  1BXIA                 55    0.155  33.102   1.433   0.004   0.514   0.481
B P  1BXIA                 56    0.080  11.395   0.201   0.004   0.138   0.858
E K  1BXIA                 57    0.664 136.667   0.618   0.018   0.019   0.964
E E  1BXIA                 58    0.761 132.912   0.058   0.016   0.005   0.979
E G  1BXIA                 59    0.614  48.338  -1.468   0.016   0.005   0.979
E D  1BXIA                 60    0.633  91.158  -1.698   0.018   0.019   0.964
E D  1BXIA                 61    0.641  92.339  -0.993   0.018   0.019   0.964
E D  1BXIA                 62    0.331  47.654  -1.743   0.018   0.019   0.964
E S  1BXIA                 63    0.260  30.425   0.704   0.053   0.005   0.942
B P  1BXIA                 64    0.134  19.000  -0.886   0.502   0.002   0.495
E S  1BXIA                 65    0.454  53.232   0.532   0.694   0.003   0.303
E G  1BXIA                 66    0.246  19.321   0.411   0.858   0.002   0.139
B I  1BXIA                 67    0.029   5.328   0.771   0.970   0.001   0.030
B V  1BXIA                 68    0.063   9.698   0.678   0.975   0.003   0.022
E N  1BXIA                 69    0.493  72.190   1.501   0.970   0.001   0.030
B T  1BXIA                 70    0.136  18.877   1.413   0.970   0.001   0.030
B V  1BXIA                 71    0.030   4.565   1.052   0.970   0.001   0.030
E Q  1BXIA                 72    0.360  64.296   1.502   0.970   0.001   0.030
E Q  1BXIA                 73    0.521  93.033   2.028   0.970   0.001   0.030
B W  1BXIA                 74    0.070  16.739   0.799   0.970   0.001   0.030
B R  1BXIA                 75    0.226  51.640   1.062   0.923   0.002   0.076
E A  1BXIA                 76    0.640  70.506   0.450   0.858   0.002   0.139
E A  1BXIA                 77    0.628  69.195   0.061   0.694   0.003   0.303
E N  1BXIA                 78    0.543  79.539   0.259   0.246   0.004   0.750
E G  1BXIA                 79    0.592  46.575  -1.707   0.016   0.005   0.979
E K  1BXIA                 80    0.317  65.269   0.810   0.016   0.005   0.979
E S  1BXIA                 81    0.514  60.217  -0.873   0.016   0.005   0.979
E G  1BXIA                 82    0.292  23.020  -1.441   0.058   0.017   0.925
B F  1BXIA                 83    0.126  25.348  -1.529   0.058   0.017   0.925
E K  1BXIA                 84    0.515 105.874   0.605   0.058   0.017   0.925
E Q  1BXIA                 85    0.701 125.163   0.469   0.058   0.017   0.925
E G  1BXIA                 86    0.890  70.051  -1.829   0.003   0.003   0.994

Explain the output.  Go back.
```

---
